# Targeting Peripheral Kappa Opioid Receptors for the Treatment of Chronic Pain: Review Article

**Published:** 2018-10-16

**Authors:** Tyler C Beck, Thomas A Dix, Carmela M Reichel

**Affiliations:** 1 Department of Drug Discovery and Biomedical Sciences, Medical University of South Carolina Campus, Charleston, SC, USA; 2 JT Pharmaceuticals, Inc., 300 West Coleman Blvd., Suite 203, Mount Pleasant, SC, USA; 3 Department of Neurosciences, Medical University of South Carolina, Charleston, SC, USA

**Keywords:** Analgesic, Kappa, Opioid, Pain, Peripheral

## Abstract

Addiction to conventional opioid pain analgesics is a major societal problem that is increasing at an alarming rate. New drugs to combat the effects of opioid abuse are desperately needed. Kappa-opioid agonists are efficacious in peripheral pain models but suffer from centrally-mediated effects. In this review, we discuss our efforts, as well as other’s efforts in developing peripheral kappa-based opioid receptor agonists that have the potential analgesic activity of opioids but do not manifest the negative side-effects of opioid use and abuse. Further, derivatives of the tetra peptide D-Phe-D-Phe-D-Nle-D-Arg-NH_2_, such as CR665, exhibit high peripheral to central selectivity in analgesic models when administered intravenously (IV); however, they are inactive when administered orally. Application of the JT Pharmaceuticals non-natural amino acid technology to CR665 produced derivatives that exhibit peripheral analgesic activity when dosed orally but do not promote CNS-based effects. Lead compound **JT09** activates the kappa-opioid receptor with EC50s in the low nM range, while agonist selectivity for kappa over other peripheral opioid receptors was >33,400 fold. Results indicate that **JT09** acts as efficacious as morphine in alleviating peripheral pain, while failing to produce undesired CNS-mediated activity. Additionally, **JT09** did not promote other CNS-mediated effects associated with morphine (addiction, sedation, dysphoria, tolerance). Thus, we propose that **JT09** has potential for development as a novel analgesic.

## INTRODUCTION

One in five people globally suffer from chronic pain [1,2]. In the United States, approximately 100 million adults are affected by chronic pain, costing our society an estimated 635 billion dollars annually [3]. Chronic pain management is cited as the most common reason Americans seek medical intervention [4]. Current pain medications in use, which are largely mu-(u-) opioid receptor agonists (MOAs), are often ineffective in the treatment of moderate to severe chronic pain and have many other significant limitations associated with the side-effects of use. Side-effects associated with MOAs including nausea, vomiting, constipation, depressed breathing, renal toxicity, thromboemolytic risk, and neurotoxicity [5]. Most notably, such compounds promote significant abuse liability and lead to the development of tolerance, thus requiring dose escalations to maintain therapeutic efficacy. It is estimated that approximately two million Americans suffer from a substance abuse disorder involving prescription pain medications [6]. In 2015, there were over 42,000 reported accidental opioid overdose deaths in the United States, which is a 600% increase since 1999 [7]. Thus, the current opioid crisis poses a significant public health problem that could be mitigated by new therapeutic approaches to pain management.

Despite the current opioid crisis, prescription of opioid analgesics continues to remain strikingly high due to the lack of development of novel classes of analgesics. To illustrate, in 2004, the peptide ziconotide (Prialt, Elan) and the small molecule pregabalin (Lyrica, Pfizer) were the first novel pain agents approved for use in the United States in over 40 years [8]. Both compounds function by targeting N-type calcium channels to inhibit neurotransmitter release at the synapse and thus blocking pain sensation; however, both have significant limitations. The administration of ziconotide requires an invasive procedure, in which a pump is implanted in the patient’s spinal cord, which significantly limits its utility. Pregabalin has been approved for only a limited number of indications, peripheral neuropathy and post-herpetic neuralgia. Both ziconotide and pregabalin demonstrate significant toxicities due to their selectivity for calcium channels, such as nausea, vomiting and neuropsychiatric disturbances. Despite their extensive side-effect profile, both drugs continue to demonstrate financial success, as ziconotide and pregabalin achieve $200 million/year and $2.7 billion/year in revenue, respectively. Pregabalin has been a “top 10 prescribed” drug for many years. All told, these figures illustrate the potential economic impact and benefit that a novel class of analgesics would provide.

**Figure 1.**
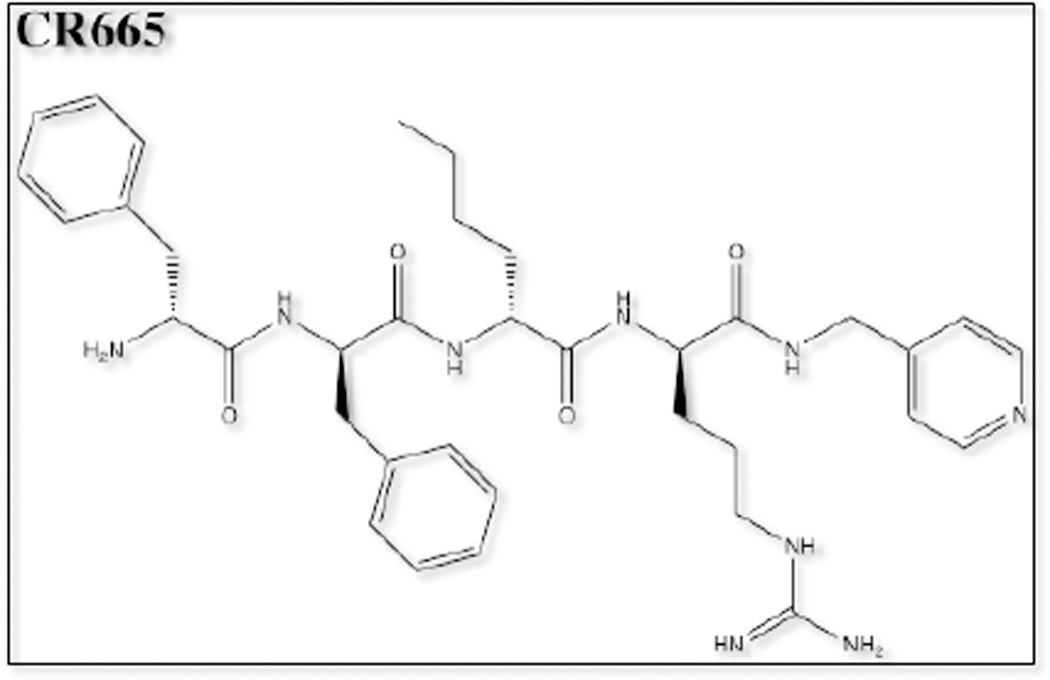
Chemical structure of CR665

**Figure 2.**
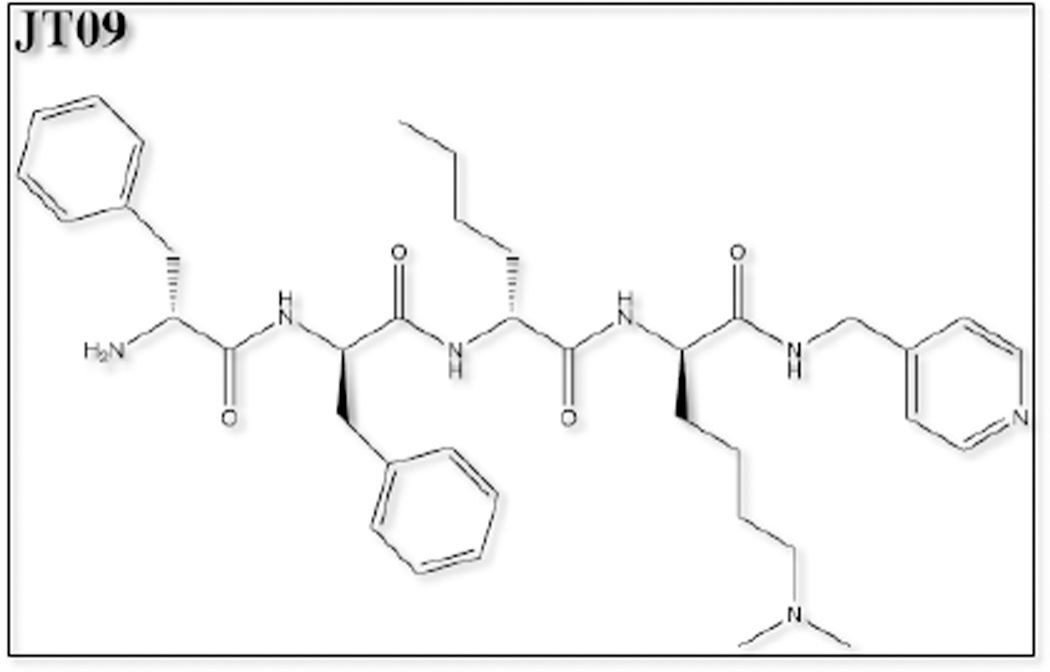
Chemical structure of JT09

**Figure 3.**
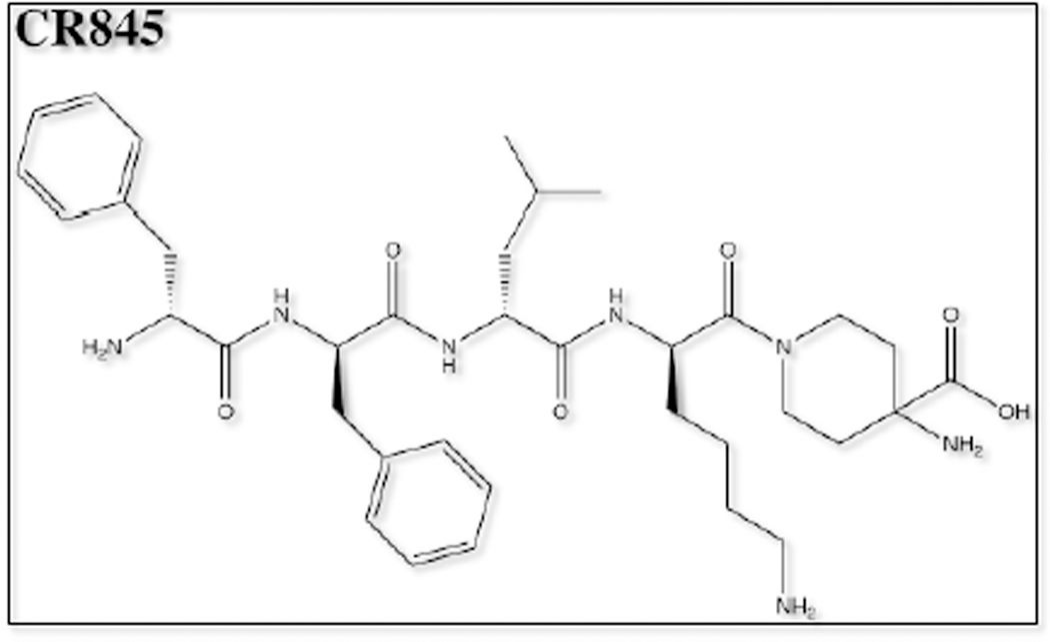
Chemical structure of CR845.

**Figure 4.**
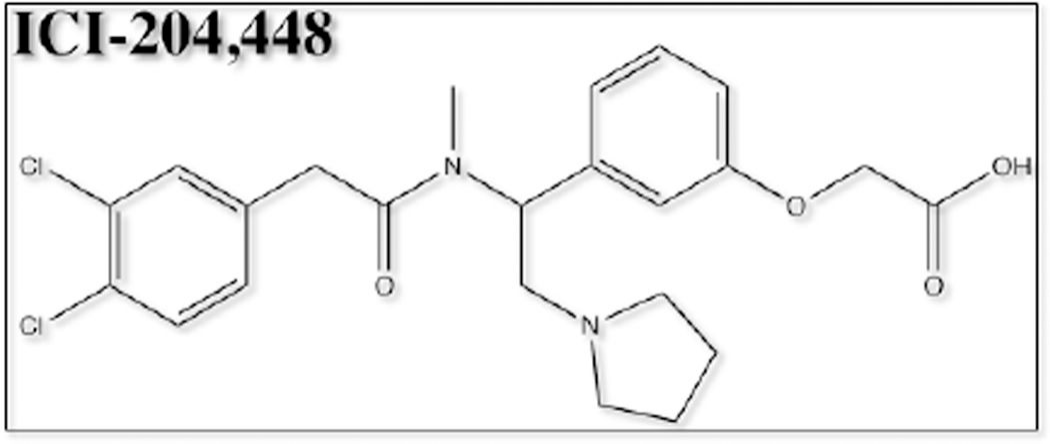
Chemical structure of ICI-204,448.

Of the opioid analgesics, kappa-opioid receptor agonists (KOAs) have demonstrated the greatest efficacy in visceral pain models. Further, peptide-derived KOAs have demonstrated a limited side-effect profile when compared to conventional opioid analgesics that target the μ-opioid receptor [9,10]. The analgesic properties of KOAs are mediated in the periphery by acting on kappa-opioid receptors (KORs); however, KOA also act centrally, leading to untoward psychoactive side effects such as dysphoria, hallucinations, sedation and psychosis [11]. The development of a peripherally-restricted KOA that does not cross the blood brain barrier (BBB) could serve as a potential broad-spectrum analgesic for chronic pain. The peptidic KOA CR665 (Figure 1), under development by Cara Therapeutics (Shelton, CT), has shown to exhibit high peripheral versus central activity and has demonstrated efficacy in the treatment of visceral and neuropathic pain without the negative side-effects of centrally acting KOAs. However, CR665 does not have significant oral bio-availability, which limits its potential use as a broad- spectrum analgesic. The JT Pharmaceutical (JT Pharma) compound JT09 (Figure 2), a second-generation compound related to CR665, is an orally active peptide analgesic that is currently being studied for the indication of chronic pain. The peripherally-restricted KOA, CR845 (Figure 3), is currently under phase three clinical trials and was found to attenuate pruritus, as well as acute and chronic pain. The drug is being dosed intravenously (i.v.) and was well tolerated in 300 patients during phase two clinical trials [9]. Another established intravenously available KOA that acts primarily in the periphery is ICI 204,448 (Figure 4), which has shown to attenuate pruritus and inflammatory pain. Additionally, when administered to rats 10 min prior to the onset of myocardial ischemia, ICI 204,448 substantially reduced infarct size, demonstrating the potential use of KOAs for the indication of myocardial infarction [12]. However, ICI 204,448 demonstrated sedative effects at doses as low as 2 mg/kg i.v., which could be explained by peripheral activation of the vagus nerve or due to centrally penetrative effects on the cerebellum [2].

In this review, we describe the characteristics of JT09 that demonstrate its potential for development as a novel, broad-spectrum, analgesic.

## METHODS AND RESULTS

Application of the JT Pharma proprietary peptide modification strategy imparts improved stability and oral availability with most peptides evaluated thus far [13]. In pharmacokinetic/pharmacodynamic (PK/PD) studies, JT09 displayed a potent kappa-opioid receptor selectivity versus the mu- and delta-opioid receptors of >33,400-fold; a peripheral-selectivity of >938-fold; an oral EC50 of 4.7 mg/kg; and an i.v. EC50 of 0.032 mg/kg [2]. These PK/PD numbers are minimal estimates, since mu- or delta- activities were not detected. It was also demonstrated that JT09 does not act as a kappa-opioid receptor antagonist or an inverse agonist.

In rat pain efficacy models, JT09 was evaluated in an acetic acid writhing assay, an established measure of peripherally mediated pain, and in a hot plate assay, a measure of centrally mediated pain. In the acetic acid writhing assay, rats were orally gavage (p.o.) with JT09 (20 mg/kg), while control rats received either morphine (10 mg/kg, intraperitoneally (i.p.)) or saline (0.2 mL, p.o.). JT09 demonstrated indistinguishable efficacy when compared to morphine in the acetic acid writhing assay for peripheral pain. In the hot plate model, rats received JT09 (20 mg/kg, p.o.), while control rats received morphine (10 mg/kg, i.p.). Rats were tested for centrally-mediated pain 20 min later by measuring the latency to paw licking behavior following placement on a 53°C hot plate. In the hot plate model, JT09 did not attenuate centrally-mediated pain, thus supporting that JT09 does not cross the BBB. Behavioral assays were performed in order to assess the side effect profile associated with JT09. Addiction is a major issue associated with conventional analgesics, such as morphine and other opioid derivatives. In order to assess the abuse liability associated with JT09, rats were studied in a self-administration model, in which rats were trained to press a lever to receive an intravenous drug infusion. Rats failed to maintain lever responding when switched from sucrose to JT09 (20 mg/kg/infusion), extinguishing lever pressing behavior within five days. This finding suggests that JT09 does not promote reward seeking behavior. When switched to cocaine (50 μg/uL/infusion), rats resumed lever pressing behavior, thus proving that the rats tested were not deficient in reward processing. In order to further assess abuse liability, a conditioned place preference model was performed, where rats would receive alternate oral doses of JT09 (20 mg/kg) and saline (0.2 mL) in their respective compartment (right or left, randomly assigned). The amount of time spent in each particular compartment was used to determine if the rats developed an association of JT09 with a particular compartment, which is a sign of addictive predilection. Results demonstrated that rats did not develop a preference for JT09 over saline, further suggesting that JT09 does not promote drug seeking behavior.

The most common cause of discontinuation of centrally-acting KOA drugs, such as U50,488, enadoline, ADL 10–0101, and ADL 10–0116, is the promotion of dysphoria and sedation [14]. In order to assess the induction of dysphoria, a forced swim assay was performed. Depressive effects are measured by the amount of time spent immobile during the last 4 min of each trial. Administration of JT09 (20 mg/kg, p.o.) did not promote immobility beyond that of saline, whereas salvinorin A (1 mg/kg, i.p.), a centrally-acting KOA, promoted significant immobility [5]. This finding suggests that JT09 does not promote dysphoria. In order to assess sedation, rats were studied in locomotor boxes to assess activity levels following the administration of either saline (0.2 mL, p.o.), morphine (10 mg/kg, i.p.) or JT09 (20 mg/kg, p.o.). Results indicated that there was a significant difference in the activity level of rats that received morphine when compared to JT09; however, the activity levels were indistinguishable when comparing rats that received JT09 and saline. This supports that JT09 does not promote sedative effects due to its high peripheral selectivity for the kappa receptor over the mu receptor. JT09 was assessed in a multiple dose study, where rats were dosed at their EC50 for 14 days, sacrificed, and then assessed via histological examination. On examination, rats administered JT09 did not demonstrate any gross or microscopic changes to the sampled tissues. This suggests that JT09 does not promote any noticeable toxicity in rats at the ED50 dose level. Further toxicity studies will be performed administering JT09 at higher dose levels.

## SIGNIFICANCE

There are many pharmacological and non-pharmacological treatments of chronic pain; however, very few treatment modalities have proven to demonstrate sustainable efficacy. In particular, mu-opioid receptor agonists (MOAs) provide sufficient analgesia acutely in many patients, yet suffer from untoward side effects, such as those mentioned above. Given the significant burden of current opioid and non-opioid medications used in pain management, there remains a significant need for a novel class of analgesics that lacks abuse liability and has a diminished side effect profile. Much like the well-known MOAs, kappa-opioid agonists (KOAs) have significant analgesic properties; however, KOAs potentiate negative side effects such as sedation, hallucinations and dysphoria, thus limiting their development as a viable therapeutic. Developing a KOA with limited CNS penetration would provide analgesic coverage against chronic pain without the aforementioned negative side effects associated with the agonism of centrally located kappa opioid receptors. Pre-clinical and clinical studies show that peptide-derived, peripherally- restricted, orally active kappa opioid agonists such as JT09, CR845, and ICI 204,448 offer promising qualities and could potentially be the solution to this problem. Further, application of the JT Pharma peptide modification strategy to CR665 imparted oral bioavailability, while enhancing the potency and PK/PD properties of the parent compound. CR845, a derivative of CR665 under development by Cara Therapeutics, demonstrated 15% oral bioavailability in Phase I clinical trials following reformulation to improve oral delivery. Given our preliminary results in pre-clinical studies, we suspect that JT09 will demonstrate significantly improved oral bioavailability without the specific need for reformulation. Additionally, JT09 does not promote sedation, whereas IC 204,448 demonstrated significant depression of locomotor activity [2].

On average, 115 people a day die from an opioid-induced drug overdose, thus emphasizing the potential ground breaking nature of developing an orally-active non-addictive analgesic with retained efficacy [7]. In comparison, 44 people died per day during the peak of the HIV/AIDs crisis, further outlining the significance of this public health epidemic. A possible solution to this issue is the peripherally-restricted kappa opioid agonist, JT09. The oral EC50 of JT09 in rats appears to be druggable when extrapolated to human doses, while retaining the efficacy of morphine. Further, we have established that JT09 does not promote CNS-mediated effects associated with centrally mediated KOAs or commonly used opioids such as morphine (i.e. sedation, dysphoria, tolerance, addiction, etc.). Thus, we propose that JT09 has potential for development as a novel analgesic.

A full report of this letter is in press at Advances in Pharmacology and Clinical Trials.
